# Androgen Deprivation Therapy for Prostate Cancer Influences Body Composition Increasing Risk of Sarcopenia

**DOI:** 10.3390/nu15071631

**Published:** 2023-03-28

**Authors:** Jolanta Korczak, Marcin Mardas, Maria Litwiniuk, Paweł Bogdański, Marta Stelmach-Mardas

**Affiliations:** 1 Department of Chemotherapy, The Greater Poland Cancer Center, 61-866 Poznan, Poland; jolanta.korczak@wco.pl; 2 Department of Gynecological Oncology, Institute of Oncology, Poznan University of Medical Sciences, 61-569 Poznan, Poland; marcin.mardas@ump.edu.pl; 3 Department of Cancer Pathology and Prevention, Poznan University of Medical Sciences, 61-866 Poznan, Poland; maria.litwiniuk@wco.pl; 4 Department of Obesity Treatment, Metabolic Disorders and Clinical Dietetics, Poznan University of Medical Sciences, 61-569 Poznan, Poland; pbogdanski@ump.edu.pl

**Keywords:** muscle mass, visceral fat, subcutaneous fat, sarcopenia

## Abstract

Computed tomography (CT) scans used in treatment response assessment in prostate cancer (PCa) patients are a useful tool for nutritional status evaluation. The aim of this study was to assess the nutritional status, including sarcopenia development based on CT scans, in PCa patients and its association with progression-free survival (PFS). Sixty-four PCa patients were included (group 1: 34 patients undergoing androgen deprivation therapy (ADT) with docetaxel due to newly diagnosed, hormone-sensitive, metastatic PCa and group 2: 30 patients with castration-resistant metastatic PCa continuing ADT therapy with enzalutamide or abiraterone acetate). Nutritional status was evaluated with anthropometrical parameters, Nutritional Risk Score (NRS), and CT scans at the L3 vertebrae. Survival analyses were performed. According to NRS, nutritional status was significantly related to PFS. In both groups, there was a significant reduction in muscle tissue (total muscle tissue and skeletal muscle index). A significant increase in the distribution of adipose tissue (subcutaneous fat, visceral fat, subcutaneous adipose tissue index, and visceral adipose tissue index) in group one was observed. Sarcopenia was diagnosed in patients but with no influence on PFS. Significant reduction in muscle mass and increase in fat mass was observed in patients treated for PCa with no impact on PFS. The NRS was related to PFS in PCa patients and associated with body composition, assessed by CT after the castration therapy. Long-term castration combined with abiraterone therapy with prednisone or enzalutamide significantly influenced muscle tissue and may lead to sarcopenia development.

## 1. Introduction

Prostate cancer (PCa) is one of the most frequently diagnosed neoplasms in men, diagnosed mostly after the age of 60 and with peak incidence after the age of 75 [1]. It can be asymptomatic for a very long time [2]. A patient’s nutritional status (i.e., malnutrition) is a factor that significantly influences therapeutic decisions and treatments for cancer. The consensus of several scientific societies (European Society for Clinical Nutrition and Metabolism (ESPEN), American Society for Parenteral and Enteral Nutrition (ASPEN), Federación Latinoamericana de Terapia Nutricional, Nutrición Clínica y Metabolismo AC (FELANPE), The Parenteral and Enteral Nutrition Society of Asia (PENSA)) at the Global Leadership Initiative on Malnutrition (GLIM) has introduced uniform criteria for the diagnosis of malnutrition [3]. The GLIM includes etiological criteria (reduced food intake and inflammation) and phenotypic criteria (weight loss, Body Mass Index (BMI), and muscle mass reduction). It was estimated that between 38 and 70% of cancer patients experience a loss of muscle mass, indicating sarcopenia development [4]. The factors responsible for age-related sarcopenia include neuromuscular degeneration, disorders of protein metabolism within the muscles, changes in hormone levels, oxidative stress, and behavioral changes [5]. The definition of sarcopenia proposed by the European Working Group on Sarcopenia in Older People (EWGSOP) and the Asian Working Group on Sarcopenia (AWGS) takes into account three criteria: low muscle strength, low muscle mass or quality, and low fitness status [6,7]. Dual Energy X-ray Absorptiometry (DXA) and Bioelectrical Impedance Analysis (BIA) are the most frequently used in sarcopenia severity assessment. However, computed tomography (CT) scans are regularly repeated in cancer patients to assess the effectiveness of the treatment. Therefore, CT scans may also be considered a very useful tool in nutritional status assessment—specifically muscle mass evaluation [8]. Sarcopenic obesity and muscle loss are poor prognostic factors in prostate cancer as they are often hidden by weight gain. In the current study, the risk of malnutrition was assessed with subjective and objective tools: the Nutritional Risk Score (NRS) 2002, GLIM (including BMI changes), and CT as a precise tool for muscle mass evaluation. The aim of the study was to assess the nutritional status, including sarcopenia development based on CT scans, in PCa patients and its association with progression-free survival (PFS).

## 2. Materials and Methods

### 2.1. Study Design and Patient Population

This is a prospective observational study conducted in the Greater Poland Cancer Centre (GPCC) between 2016 and 2021. Qualification for oncological treatment was based on the current guidelines of the Polish Society of Clinical Oncology (PTOK) [9], the European Society of Urology (EAU) [10], and the European Society for Medical Oncology (ESMO) [11]. The study was approved by the Bioethics Committee (No: 1134/2016) at the Poznan University of Medical Science, Poland. The research was conducted in accordance with the Helsinki Declaration. Patients were recruited by oncologists during initial consultation. Inclusion criteria were as follow: prostate adenocarcinoma confirmed by histopathology, and disease stage IV—the presence of distant metastases. Treatment groups were as follows. Group 1: patients undergoing androgen deprivation therapy (ADT) with docetaxel due to newly diagnosed, hormone-sensitive, metastatic prostate cancer. Group 2: Patients with castration-resistant metastatic prostate cancer who continued ADT therapy in combination with enzalutamide or abiraterone. Group 2 patients also had tomographic examinations available and consented to participate in the study. Exclusion criteria: coexistence of a second cancer or another disease that is a contraindication to the planned treatment, cancer cachexia, refusal to participate in the study, uncontrolled brain metastases, known history of HIV (HIV 1/2 antibodies), known active hepatitis B or C, diagnosis of immunodeficiency or systemic immunosuppressive therapy, uncontrolled cardiovascular disease, active drug or alcohol abuse, legal incompetence, and limited legal competence.

### 2.2. Methods

The risk of malnutrition was assessed according to the Nutritional Risk Score (NRS) 2002 scale. In accordance with ESPEN recommendations, patients who obtained an NRS score of 3 or more were encouraged to start a nutritional intervention, i.e., dietary consultation and introduction of Oral Nutritional Support (ONS) (125 mL including 18 g of protein and 300 kcal) [12,13,14,15]. Standardized dietary counseling was conducted by qualified nutritionists and was structured according to relevant topics linked to healthy lifestyle and good sources of food rich in protein and energy, i.e., polyunsaturated fatty acids. All patients had the opportunity to explain their “nutritional” story after they detected that their body weight started to decrease. Participants were advised to use well-balanced diets based on their food preferences (approx. 18% of energy from protein and 25–30% from fat). The goals of the dietary modification included increasing energy and protein intake to meet the energy requirement and to improve patients’ nutritional status. Patients were educated on recommended techniques that should be used during meal preparation, hygienic eating, etc. To determine malnutrition, patients were also analyzed according to the GLIM criteria [3]. According to the GLIM guidelines, all patients met at least one etiological criterion—disseminated neoplastic disease. Phenotypic criteria were based on the analysis of changes in body weight, BMI, and measurement of muscle mass in a CT scan of the L3 vertebrae.

Total Prostate-Specific Antigen (PSA) concentration (ng/mL) was determined on a Roche cobas e801 analyzer using the ECLIA (electrochemiluminescence) method based on streptavidin-coated microparticles, biotin-labeled anti-PSA antibodies, and ruthenium-Ru (bpy) complex _3_^2^ + (Trus (2.2 ‘bipyridyl) ruthenium (II) -complex). The PSA was measured at each visit. The PSA Doubling Time was used for the evaluation of dynamic changes. An increase in PSA concentration must meet certain criteria to determine the biochemical progression of the disease [16,17]. To find castration resistance, it is necessary to determine disease progression at castration testosterone levels, and a three-fold PSA increase by more than 50% from the lowest value achieved during treatment—nadir—was considered as a biochemical progression in examinations performed at intervals of at least one week, with a nominal PSA concentration > 2 ng/mL [18]. To find castration resistance, it is necessary to determine disease progression at castration testosterone levels. Quantification of testosterone concentration (ng/mL) in plasma was performed by the ECLIA method on the cobas e801 analyzer from Roche using streptavidin-coated microparticles.

**Figure 1:**
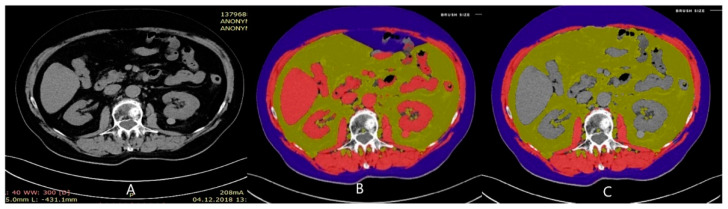
Measurement of the muscle and visceral and subcutaneous fat tissue areas at the L3 vertebrae with CoreSlicer: (**A**): naïve image; (**B**): automatically outlined areas; (**C**): manual correction.

The CT scans were performed on a 128-row Siemens Somatom Sensation AS PLUS (Adaptive Scanning) apparatus; Ultravist 370 or Visipaque 320 contrasts were used for the study. Progression according to the international criteria for evaluating the response to treatment in solid tumors—the Response Evaluation Criteria in Solid Tumors (RECIST 1.1) were used [19]. The CT scans were performed at the baseline and when biochemical progression occurred. Measurement of the muscle tissue area at the L3 vertebrae was performed using a free automatic online tool—CoreSlicer.com. Software [20] automatically measured the area of total muscle tissue (TM), subcutaneous fat (SF) and visceral fat (VF). Then, if necessary, when the software incorrectly classified anatomical structures in individual patients (e.g., kidneys or large intestine as muscle tissue), the contours of anatomical structures were manually corrected (Figure 1). The obtained results in cm^2^ were recorded, and the obtained value was used to calculate the Skeletal Muscle Index (SMI) parameter according to the following formula: SMI(cm^2^/m^2^) = TM(cm^2^)/Height(m^2^). The obtained value was corrected in relation to the patient’s height, obtaining the Subcutaneous Adipose Tissue Index (SATI) with the use of the following formula: SATI (cm^2^/m^2^) = SF(cm^2^)/Height^2^(m^2^), and the Visceral Adipose Tissue Index (VATI) with the use of the following formula: VATI (cm^2^/m^2^) = VF(cm^2^)/Height^2^(m^2^). By means of changes in SMI, SATI, and VATI, changes in the scope of tissue composition of the body during therapy were visualized. Based on the obtained value of the SMI index, sarcopenia was diagnosed in study patients. For sarcopenic men, the most commonly recognized SMI value is 53 cm^2^/m^2,^ and it was used as the cut-off point [4,21]. The cut-off point for the SMI for sarcopenia is lower in patients with a BMI < 25 kg/m^2^ at 43 cm^2^/m^2^ [21]. The criteria for the diagnosis of sarcopenic obesity included BMI > 30 kg/m^2^ and SMI < 53 cm^2^/m^2^.

### 2.3. Statistical Analysis

For quantitative variables, descriptive statistics were used, such as arithmetic mean, range, and standard deviation. For qualitative variables, an assessment of the number of percentages was used. Depending on the data distribution, when assessing changes before and during treatment, the following test was used: normal distribution (when k = 2 Student’s *t*-test for related and unrelated variables or k > 2 ANOVA and distribution inconsistent with the normal distribution), Friedman’s test for related variables, and Kruskal-Wallis test for unrelated variables. Contingency table analysis, including Relative Risk (RR) was performed with the Exact Fisher’s Test. The survival analysis was calculated with the log-rank test (Mantel-Cox) and presented with a hazard ratio (data were adjusted for age).

Statistical analyses were performed using STATISTICA 13.3 (TIBICO Software Inc., Palo Alto, CA, USA, 2017) and GraphPad Prism 5.01 (GraphPad Software).

## 3. Results

The characteristics of the study population are presented in Table 1. Median observation time was 12.33 months (range: 5.4–26 months). During the follow-up period, 10 deaths in group 1 and 6 deaths in group 2 of patients were noticed. According to the collected data, the patients differed significantly only in terms of body height. Based on BMI, the percentage of patients who developed underweight, overweight, or obesity was similar in both groups. The pathomorphological assessment of PCa according to the Gleason score also did not differ significantly between the groups. In both groups, most patients had bone and lymph node metastases, and only 3% of patients in group 1 had parenchymal organ metastases. Following the NRS interpretation, 20% and 38% of patients in groups 1 and 2, respectively, were qualified for dietary consultation. All of them received dietary modification plans, and approx. 50% in both groups received ONS. None of them required enteral or parenteral nutrition. WHO performance status showed that the majority of patients presented symptoms but were able to perform light work. However, 1/4 of the patients in group 1 were in worse condition. The history of treatment and the presence of comorbidities is presented in Table 2.

The analysis of changes in body composition based on the measurements obtained from CT (TM, SF, and VF) and the SMI, SATI, and VATI are presented in Table 3. Significant changes were found in TM, SF, VF, SMI, SATI and VATI in group 1 of patients with hormone-sensitive metastatic prostate cancer and in TM and SMI in group 2 of patients with castration-resistant metastatic prostate cancer who continued ADT therapy in the combination with enzalutamide or abiraterone acetate. In both groups, there was a significant reduction in muscle tissue (TM and SMI), which suggests that the loss of muscle mass occurs both at the initial stage of treatment and after long-term castration. Significant increase in the distribution of adipose tissue (SF, VF, SATI, and VATI) in group 1 of patients was observed. These changes suggest that initiation of castration therapy in combination with chemotherapy with docetaxel leads to an increase in body fat. Among patients from group 2, an increase in the amount of adipose tissue (SF, VF, SATI, VATI) was also observed; however, these changes were not statistically significant, which suggests that the greatest increase in adipose tissue occurs immediately after castration. A subgroup of patients at high risk, according to NRS, was also presented. All of the analyzed parameters significantly changed with TM and SMI loss and SF, VF, SATI and VATI increase.

**Figure 2:**
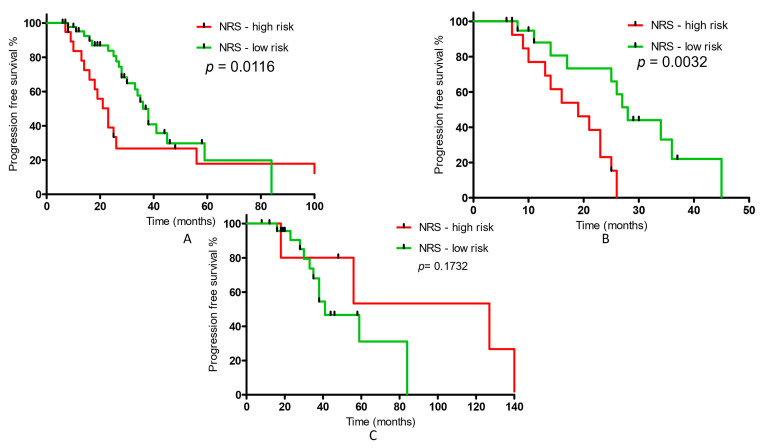
The distribution of progression-free survival for (**A**) all patients with prostate cancer, (**B**) patients undergoing ADT therapy with docetaxel due to newly diagnosed, hormone-sensitive, metastatic prostate cancer, (**C**) patients with castration-resistant metastatic prostate cancer, continuing ADT therapy in combination with enzalutamide or abiraterone acetate in relation to changes in nutritional status (according to the NRS).

Patients at low (*n* = 45) and high risk (*n* = 19) of malnutrition, according to NRS, had a median survival time of 36 and 23 months, respectively (*p* = 0.0116; HR: 1.835; 95% CI: 0.861; 3.912). In group 1, patients at low (*n* = 21) and high risk (*n* = 13) of malnutrition, according to NRS, had a median survival time of 28 and 19 months, respectively (*p* = 0.0032; HR: 4.563; 95% CI: 1.664; 12.51). In group 2, the results were not statistically significant; however, only 6 patients were in NRS high-risk group (*p* = 0.1732; HR: 0.4262; 95% CI: 0.1249; 1.454) (Figure 2).

The influence of selected body composition parameters assessed by computed tomography on progression-free survival is presented in Table 4. Based on the values of BMI and SMI, sarcopenia was diagnosed in the study population (*n* = 16 in group 1 and *n* = 14 in group 2). During the treatment, the percentage of patients with sarcopenia increased in both groups but was statistically significant in group 2 only. Nevertheless, these results did not significantly translate into survival in either group 1 or group 2 (Figure S1A,B).

**Figure 3:**
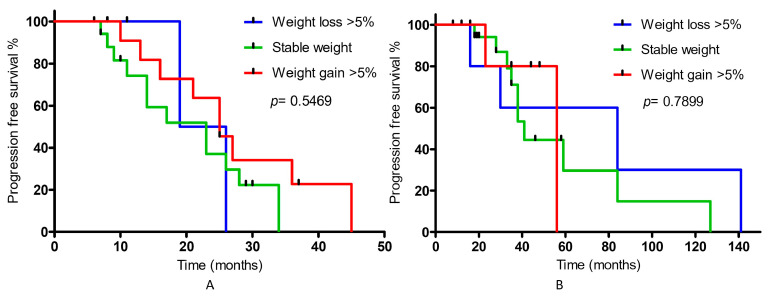
Progression-free survival for patients (**A**) undergoing ADT therapy with docetaxel due to newly diagnosed, hormone-sensitive, metastatic prostate cancer, (**B**) with castration-resistant metastatic prostate cancer, continuing ADT therapy in combination with enzalutamide or abiraterone acetate in relation to body weight changes.

For all patients with BMI >30 kg/m^2^ (*n* = 28), the median survival time was 35 months, and for patients with BMI <30 kg/m^2^, the median survival time was 30 months; however, the differences were not statistically significant (*p* = 0.8864). Similarly, there were no statistically significant differences in either group. No significant effect on changes in muscle mass (stable and 10% decrease) was observed (median survival in each group: 25 months) (Figure S3). For patients with a 10% increase in SF (group 1: *n* = 16; group 2: *n =* 8), the median survival time in group 1 was 25 months, while for patients with a stable SF content, it was 23 months; however, the differences were also not statistically significant. In the case of group 2, there was also no significant effect of changes in SF on PFS, but the median survival for patients with a 10% increase was 59 months, and for those with a stable content, the median survival was 56 months (Figure S4). Similarly, in patients with a 10% increase in VF (group 1: *n* = 15; group 2: *n* = 12), there was no significant effect on disease PFS in individual groups of patients. The median survival time in group 1 of patients with a 10% increase was 21 months, while for patients with stable VF content, it was 26 months. In the case of group 2, the median survival for patients with a 10% increase was 56 months, and for those with a stable content, it was 38 months (Figure S5). When analyzing the median value of SATI (72 as the cut-off point) (group 1: n = 12; group 2: n = 19), there was no significant effect on survival for patients with higher values (SATI > 72; group 1—median survival: 26 months; group 2—median survival: 56 months) and lower (SATI < 72; group 1—median survival: 23 months; group 2—median survival: 141 months) (Figure S6). There was also no significant effect of the VATI on survival, taking the median (101) as the cut-off point for the analyzed group of patients (VATI > 101; group 1—median survival: 26 months; n = 13; group 2, median survival: 59 months; n = 17) and lower (VATI < 101; group 1—median survival: 23 months; group 2—median survival: 56 months) (Figure S7). When analyzing changes in body weight in 3 subgroups of patients (patients who lost weight (group 1: *n* = 6; group 2: *n* = 5), kept it at a stable level (group 1: *n* = 17; group 2: *n* = 20), and patients who noticed weight gain (group 1: *n* = 11; group 2: *n* = 5)), no significant association between PFS and weight change was found (Figure 3).

## 4. Discussion

The nutritional status assessed by the NRS scale was significantly related to PFS and associated with changes in several body composition parameters (SF, VF, SATI, and VATI), assessed by CT after castration inclusion. Castration with abiraterone therapy with prednisone or enzalutamide also significantly reduced muscle tissue. Based on CT scans, a tendency to develop sarcopenia in the studied population of patients was observed, although it was not significantly associated with PFS.

Primary sarcopenia develops as a result of the aging process, and secondary sarcopenia is a result of a chronic inflammatory state. The presence of sarcopenia affects the survival prognosis in multiple health conditions that are commonplace in the elderly, including cancer. It is worth mentioning that sarcopenic patients have poorer tolerance to cancer therapies, with a greater incidence of complications, chemotherapy toxicity, and perioperative problems. Moreover, sarcopenia can exist in isolation or as a component of cancer cachexia. There is considerable overlap in the pathophysiology of cachexia and sarcopenia development. The key differentiating feature of cachexia is a hypercatabolic state and a negative protein energy imbalance, which is difficult to reverse. The cancer cachexia is described as loss of total body weight, and sarcopenia as specific loss of lean muscle mass. Finally, sarcopenic obesity also represents a poor prognosis in cancer patients [22]. Assessment of body composition changes is often underestimated, especially in overweight or obese patients [23]. A significant relationship has already been demonstrated between the value of the NRS score and the response to treatment, where the OS in patients diagnosed with various types of solid tumors (including gastric cancer and lung cancer) was assessed [24,25]. This is consistent with the current study. Among patients with metastatic PCa, a significant effect on PFS in patients from group 1 was proved. Owen et al. [26] examined the influence of adiposity on muscle health in men treated with ADT for PCa and showed that muscle mass, size, and strength are compromised in men treated with ADT after accounting for their increased adiposity or body size. Different studies confirmed [22,23,27,28] that PCa therapy is associated with an increase in adipose tissue. Naturally, in patients without a cancer diagnosis, this increase stabilizes around the age of 70, while castration treatment causes a constant increase in SF at the level of about 11–13% [27]. Nevertheless, the data on changes in VF are inconsistent [27,29,30] and indicate, on the one hand, a stable VF volume during ADT, and, on the other hand, an increase in VF (even up to 22%) during 50-week castration therapy [27]. The current study confirmed significant changes in the distribution of adipose tissue (SF, VF, SATI, and VATI) in group 1 patients who had an increase in both SF and VF, which suggests that the initiation of castration therapy in combination with docetaxel chemotherapy affects the growth of adipose tissue. It has to be mentioned that the SF and VF fulfill various endocrine functions and are characterized by different secretion of adipokines and lipolytic activity [28]. In our study, both groups showed an increase in SF and VF parameters. These results confirm the nature of the continuous increase in adipose tissue in patients undergoing ADT [27]. Moreover, Ebadi et al. [28] showed that a high SATI index (> 50 cm/m^2^) is associated with a better prognosis in patients with various types of cancer. After the initiation of castration therapy in patients with hormone-sensitive prostate cancer (group 1), the increase in adipose tissue was dynamic and slow in the castration-resistance phase (group 2). On this basis, it can be assumed that the addition of new hormonal drugs does not significantly affect the changes in adipose tissue. Nevertheless, there was no significant association between SF, VF, SATI, VATI, and PFS. However, a trend was observed for a longer median PFS in patients with a 10% increase in SATI. Strorer et al. [31] indicated that adverse effects of ADT on muscle function, physical performance, and body composition occur shortly after the onset of ADT and tend to persist and worsen over time. In the current study, the measurement of TM in both groups of patients showed its reduction during the therapy. It was pointed out by Rinninella et al. [32] that low muscle mass, assessed by L3 CT-scan, affects almost 1/3 of gastric cancer patients at diagnosis and acts as a negative prognostic marker for many clinical outcomes. This is consistent with the current study, where a significant decrease in TM was observed in the study population of PCa patients. The changes in TM index normalized with height (SMI) were also statistically significant. The fact that in group 2, the values of TM and SMI were slightly higher may suggest that the addition of the new hormonal drugs (abiraterone or enzalutamide) to long-term ADT may further aggravate the loss of muscle mass. Nevertheless, the changes in TM and SMI did not significantly affect the PFS.

It is worth mentioning that the high incidence of sarcopenia in patients with PCa (30–80%) [29,33] is observed in the clinical setting. It was suggested by Casirati et al. [34] that the prevalence of data on malnutrition expressed as weight loss is more in agreement with those of sarcopenia recognized using CT. This is consistent with our observation and obtained results in terms of TM and SMI changes. However, the analysis of the relationship between PFS and the presence of sarcopenia assessed in CT scans in both groups did not show a significant relationship. Similarly, in the study by Stangl-Kramser et al. [29], a significant influence of changes in body composition parameters on key parameters for assessing treatment response, overall survival (OS), and PFS was not confirmed. As was pointed out by de Rooy et al. [35], a better understanding of the regulation of muscle mass and function by androgens may have implications not only for PCa patients but also for age-associated sarcopenia in the general population [31]. A study by Miyake et al. [36] assessing the effect of the time to onset of castration resistance and its impact on OS has shown that when this time is over 18 months, patients have significantly longer survival. Similarly, in the current study, among the study patients from group 2 (patients with castration-resistant metastatic prostate cancer who continued ADT therapy in combination with enzalutamide or abiraterone acetate), the majority (90%) had a longer time (more than 18 months) to become castration resistant, which indicates that patients were characterized by slow disease progression. The study by Maeda et al. [37] suggested that PFS is a potential surrogate endpoint of OS in clinical trials for patients with PCa. Similarly, PFS was indicated as a potential surrogate endpoint for castration-resistant PCa and locally advanced PCa.

This study has several limitations. It was not possible to calculate OS due to the low number of deaths noted in the study population. As the sarcopenia was assessed only with the use of CT scans, the proposed criteria by EWGSOP and AWGS for sarcopenia diagnosis were not fulfilled. Firm conclusions cannot be drawn as the study population number was low.

## 5. Conclusions

The NRS is related to PFS in prostate cancer patients and is associated with body composition assessed by computed tomography after castration therapy. Long-term castration combined with abiraterone therapy with prednisone or enzalutamide significantly influenced muscle tissue and may lead to sarcopenia development.

## Figures and Tables

**Table 1:** Characteristics of the study patients (*n* = 64).

Analyzed Parameter	Group 1 (*n* = 34)		Group 2 (*n* = 30)		*p*-Value
	Mean	SD	Mean	SD	
Age [year]	68.6	7.1	72.0	7.5	0.0612
Body weight [kg]	87.6	17.8	85.8	15.1	0.9571
Body height [cm]	174.9	6.3	170.7	6.7	0.001
BMI * [kg/m^2^]	28.5	4.9	29.3	4.4	0.5316
BMI categories [%]					
Underweight	3		0		0.3441
Normal	20		19		
Overweight	35		37		
Obesity	42		44		
Gleason’s score	Median: 8	Range: 5–9	Median:7	Range: 4–9	
					0.5641
GS ≤ 6 (no)	1		9		
GS 7	11		14		0.008
GS 8–10	22		7		
Metastasis sites %:					0.3318
Bones	94		70		
Lymph nodes	74		53		
Parenchymal organs	3		0		
WHO scale [%]					<0.001
0	6		100		
1	70				
2	24				
NRS [%] *					0.005
0–2	80		62		
≥3	20		38		

* BMI—Body Mass Index, NRS—nutritional risk score.

**Table 2:** The medical history and the treatment method of the study population (*n* = 64).

Medical History	Group 1 (*n* = 34)	Group 2 (*n* = 30)
Current radical treatment [%]:		
Lack	88.2	23.3
Radiotherapy	11.8	63.3
Prostatectomy	0	13.3
The presence of additional diseases [%]:		
Diabetes	26.5	16.6
Hypertension	61.8	66.7
Ischemic heart disease	14.7	46.76
Other: (thyroid disease, gout, glaucoma, depression, hepatitis, cardiac arrhythmias, stroke, chronic obstructive pulmonary disease, renal failure, degenerative spine disease, radiation proctitis, valvular disease)	44.1	40.0
Qualification for therapy [%]:		
Docetaxel 75 mg/m^2^	79.5	-
Docetaxel 50 mg/m^2^	20.5	-
Enzalutamide	-	30
Abiraterone with Prednisone	-	70

**Table 3:** Changes in selected parameters assessed by computed tomography in the study patients with prostate cancer (*n* = 64).

	Baseline		Progression		
Analyzed Parameter	Median	SD	Median	SD	*p*-Value
Group 1 (*n* = 34)					
TM [cm^2^]	164.2	31.4	153.4	28.8	<0.0001
SF [cm^2^]	202.4	103.2	237.8	120.1	0.0115
VF [cm^2^]	273.8	134.6	308.9	130.6	0.0209
SMI [cm^2/^m^2^]	54.01	9.85	50.4	9.61	<0.0001
SATI [cm^2/^m^2^]	66.47	33.36	78.2	39.2	0.0088
VATI [cm^2/^m^2^]	90.40	44.2	101.7	42.6	0.0191
Group 2 (*n* = 30)					
TM [cm^2^]	151.6	25.2	138.3	20.6	<0.0001
SF [cm^2^]	250.9	86.3	267.8	115.0	0.1625
VF [cm^2^]	321.8	113.0	326.5	114.7	0.2966
SMI [cm^2/^m^2^]	52.0	8.311	47.4	6.46	<0.0001
SATI [cm^2/^m^2^]	86.1	29.7	92.01	40.2	0.1591
VATI [cm^2/^m^2^]	110.2	37.9	112.4	39.89	0.211
Subgroup with NRS ≥ 3 (*n* = 19)					
TM [cm^2^]	133.7	33.2	129.2	30.5	0.0017
SF [cm^2^]	156.0	67.9	183.4	73.2	0.0187
VF [cm^2^]	192.1	126.4	238.6	111.0	0.0075
SMI [cm^2/^m^2^]	44.3	9.84	43.4	8.81	0.0012
SATI [cm^2/^m^2^]	54.2	24.7	62.4	25.6	0.0187
VATI [cm^2/^m^2^]	70.6	40.3	87.3	36.1	0.0084

TM—total muscle, SF—subcutaneous fat, VF—visceral fat, SMI—Skeletal Muscle Index, SATI—the Subcutaneus Adipose Tissue Index, VATI—the Visceral Adipose Tissue Index.

**Table 4:** The influence of selected body composition parameters assessed by computed tomography on progression-free survival.

Comparisons	Hazard Ratio (95% CI)	
	Group 1	Group 2
Sarcopenia vs. no sarcopenia	1.424 (0.5974–3.396)	0.7539 (0.2615–2.174)
BMI < 30 vs. BMI > 30 kg/m^2^	1.159 (0.4732–2.838)	0.6522 (0.2203–1.930)
Muscle mass loss vs. stable muscle mass	1.363 (0.4850–3.830)	0.9389 (0.3202–2.753)
Subcutaneous fat increase vs. stable subcutaneous fat	1.018 (0.4220–2.456)	1.385 (0.3802–5.044)
Visceral fat increase vs. stable visceral fat	1.712 (0.7100–4.129)	1.132 (0.3488–3.676)
SATI < 72 vs. SATI > 72	1.218 (0.5002–2.968)	0.8278 (0.2642–2.593)
VATI < 101 vs. VATI > 101	1.863 (0.7842–4.424)	0.7092 (0.2376–2.117)

## Data Availability

Data will be available after the initial Medical Board of the Chemotherapy Department agreement via direct contact by jolanta.korczak@wco.pl.

## Supplementary Materials

The following supporting information can be downloaded at: https://www.mdpi.com/article/10.3390/nu15071631/s1, Figure S1: Progression-free survival distribution for patients A. undergoing ADT therapy with docetaxel due to newly diagnosed, hormone-sensitive, metastatic prostate cancer B. with castration-resistant metastatic prostate cancer, continuing ADT therapy in combination with enzalutamide or abiraterone acetate in relation to sarcopenia from 1st computed tomography; Figure S2: The distribution of progression-free survival for A. all patients with prostate cancer B. for patients undergoing ADT therapy with docetaxel due to newly diagnosed, hormone-sensitive, metastatic prostate cancer C. with castration-resistant metastatic prostate cancer, continuing ADT therapy in combination with enzalutamide or abi-raterone acetate in relation to changes in the BMI index, Figure S3: Progression-free survival distribution for patients A. undergoing ADT therapy with docetaxel due to newly diagnosed, hormone-sensitive, metastatic prostate cancer B. with castration-resistant metastatic prostate cancer, continuing ADT therapy in combination with enzalutamide or abiraterone acetate in relation to muscle mass changes. Figure S4: Progression-free survival distribution for patients A. undergoing ADT therapy with docetaxel due to newly diagnosed, hormone-sensitive, metastatic prostate cancer B. with castration-resistant metastatic prostate cancer, continuing ADT therapy in combination with enzalutamide or abiraterone acetate in relation to SF changes, Figure S5: Progression-free survival distribution for patients A. undergoing ADT therapy with docetaxel due to newly diagnosed, hormone-sensitive, metastatic prostate cancer B. with castration-resistant metastatic prostate cancer, continuing ADT therapy in combination with enzalutamide or abiraterone acetate in relation to VF changes. Figure S6: Progression-free survival distribution for patients A. undergoing ADT therapy with docetaxel due to newly diagnosed, hormone-sensitive, metastatic prostate cancer B. with castration-resistant metastatic prostate cancer, continuing ADT therapy in combination with enzalutamide or abiraterone acetate in relation to SATI changes. Figure S7: Progression-free survival distribution for patients A. undergoing ADT therapy with docetaxel due to newly diagnosed, hormone-sensitive, metastatic prostate cancer B. with castration-resistant metastatic prostate cancer, continuing ADT therapy in combination with enzalutamide or abiraterone acetate in relation to VATI changes.
