# Supplementary material for: Androgen Deprivation Therapy for Prostate Cancer Influences Body Composition Increasing Risk of Sarcopenia
Source: Nutrients. 2023 Mar 28;15(7):1631. doi: 10.3390/nu15071631 (PMC10096521; doi:10.3390/nu15071631)
Supplement: Supplementary file 1 [file nutrients-15-01631-s001.zip › nutrients-2266489-supplementary.pdf]

Supplementary file

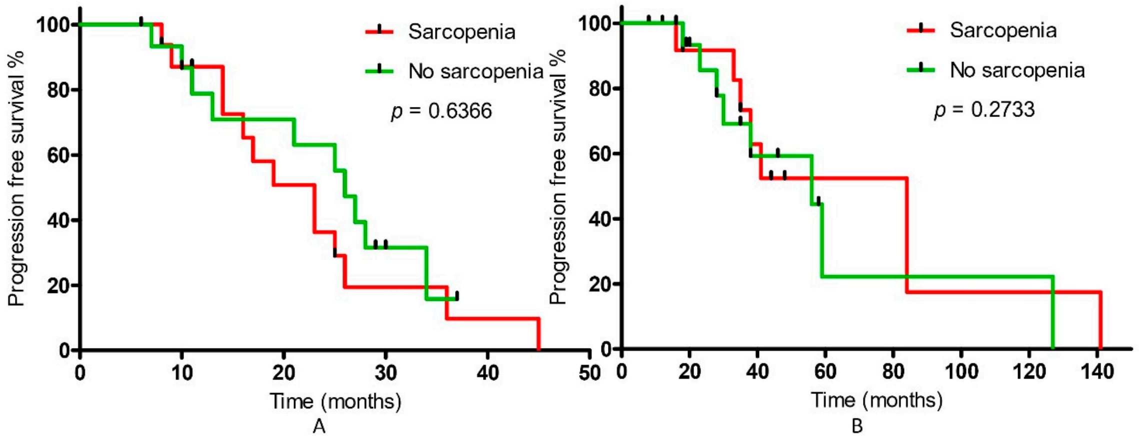

Figure S1 Progression-free survival distribution for patients A. undergoing ADT therapy with docetaxel due to newly diagnosed, hormone-sensitive, metastatic prostate cancer B. with castration-resistant metastatic prostate cancer, continuing ADT therapy in combination with enzalutamide or abiraterone acetate in relation to sarcopenia from 1st computed tomography

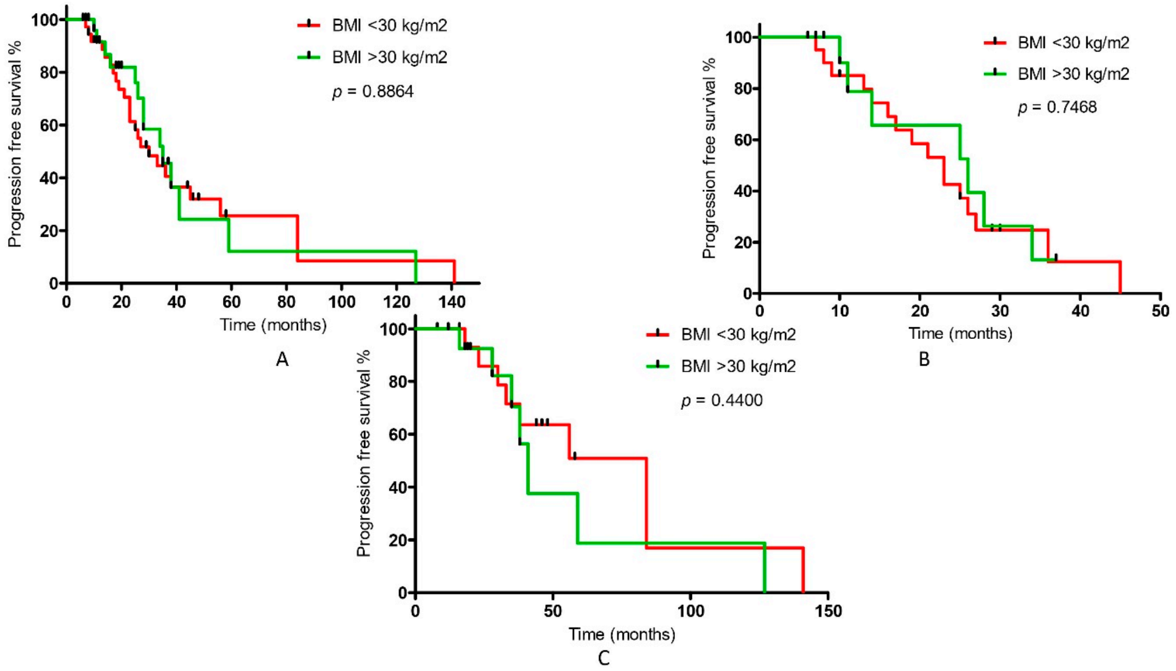

Figure S2 The distribution of progression-free survival for A. all patients with prostate cancer B. for patients undergoing ADT therapy with docetaxel due to newly diagnosed, hormone-sensitive, metastatic prostate cancer C. with castration-resistant metastatic prostate cancer, continuing ADT therapy in combination with enzalutamide or abi-raterone acetate in relation to changes in the BMI index

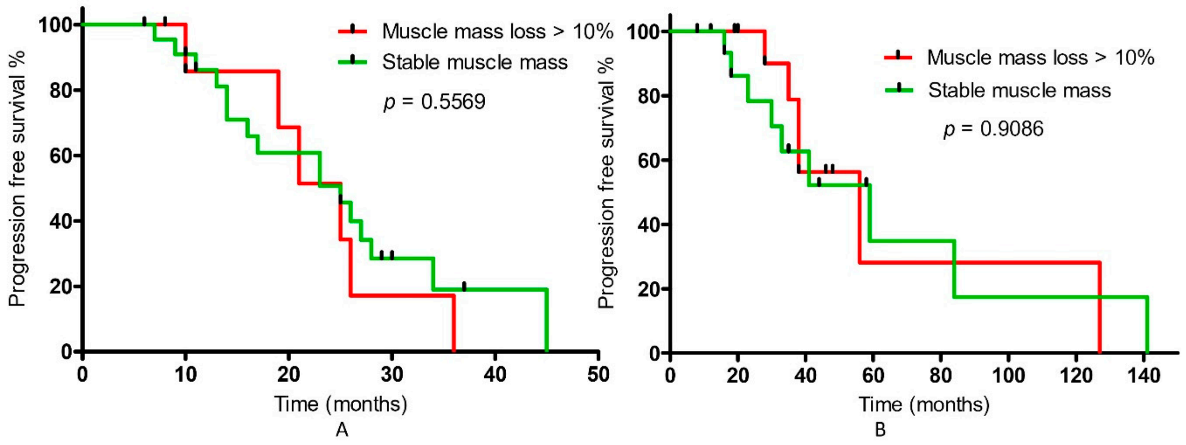

Figure S3 Progression-free survival distribution for patients A. undergoing ADT therapy with docetaxel due to newly diagnosed, hormone-sensitive, metastatic prostate cancer B. with castration-resistant metastatic prostate cancer, continuing ADT therapy in combination with enzalutamide or abiraterone acetate in relation to muscle mass changes.

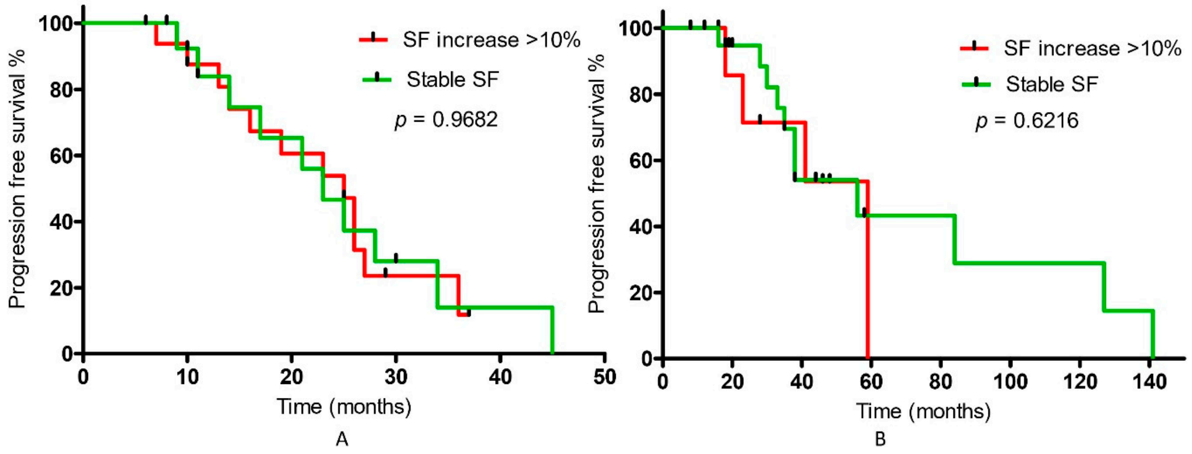

Figure S4 Progression-free survival distribution for patients A. undergoing ADT therapy with docetaxel due to newly diagnosed, hormone-sensitive, metastatic prostate cancer B. with castration-resistant metastatic prostate cancer, continuing ADT therapy in combination with enzalutamide or abiraterone acetate in relation to SF changes

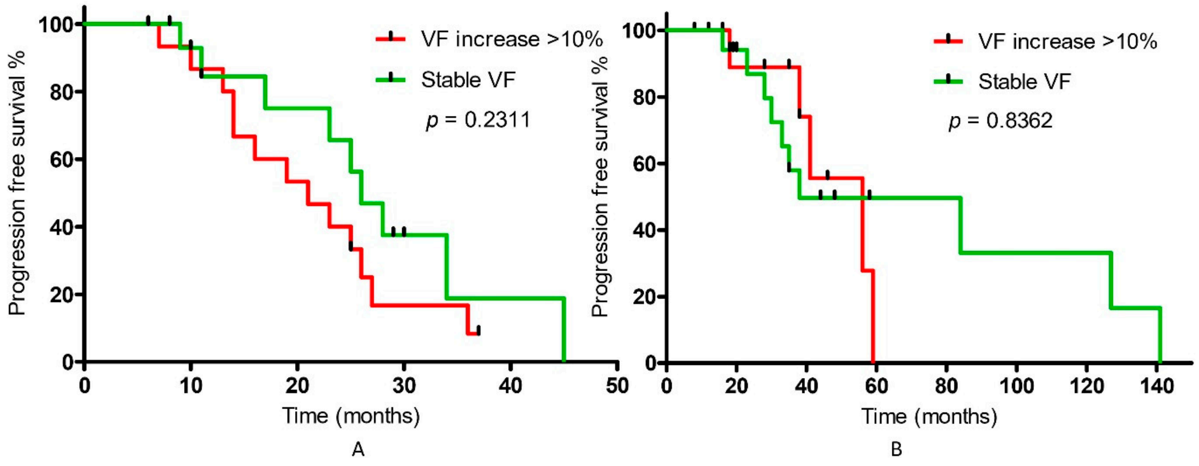

Figure S5 Progression-free survival distribution for patients A. undergoing ADT therapy with docetaxel due to newly diagnosed, hormone-sensitive, metastatic prostate cancer B. with castration-resistant metastatic prostate cancer, continuing ADT therapy in combination with enzalutamide or abiraterone acetate in relation to VF changes.

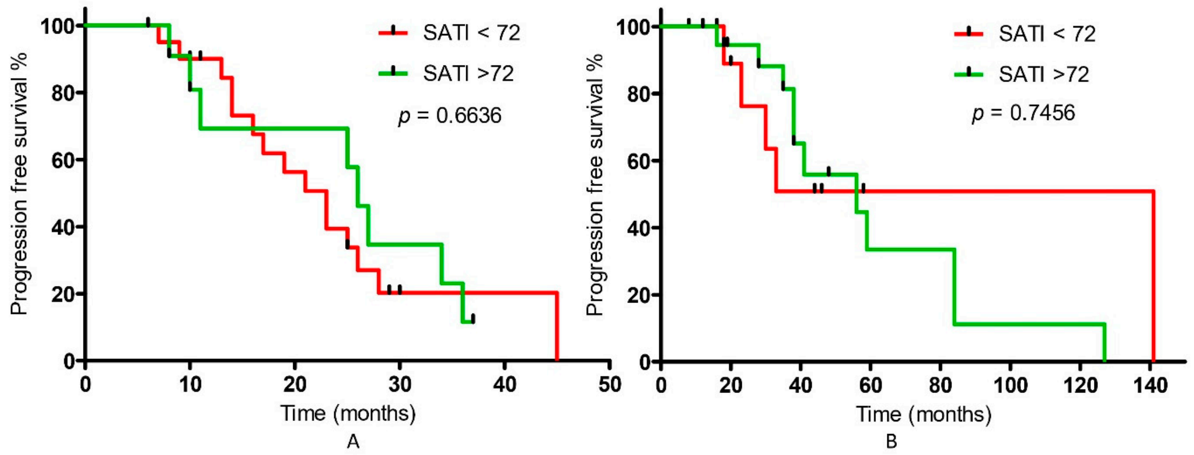

Figure S6 Progression-free survival distribution for patients A. undergoing ADT therapy with docetaxel due to newly diagnosed, hormone-sensitive, metastatic prostate cancer B. with castration-resistant metastatic prostate cancer, continuing ADT therapy in combination with enzalutamide or abiraterone acetate in relation to SATI changes.

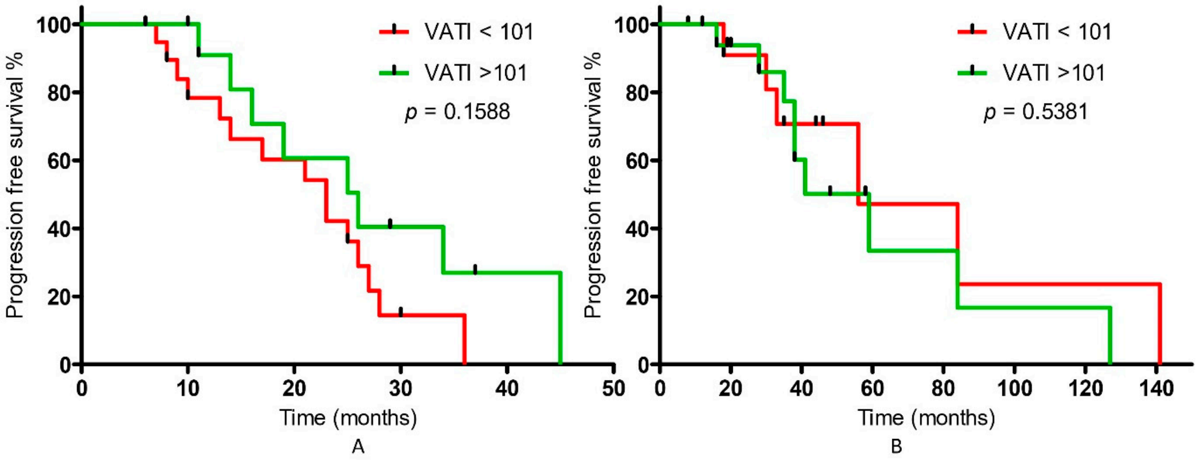

Figure S7 Progression-free survival distribution for patients A. undergoing ADT therapy with docetaxel due to newly diagnosed, hormone-sensitive, metastatic prostate cancer B. with castration-resistant metastatic prostate cancer, continuing ADT therapy in combination with enzalutamide or abiraterone acetate in relation to VATI changes.
